# Genome-Wide Runs of Homozygosity, Effective Population Size, and Detection of Positive Selection Signatures in Six Chinese Goat Breeds

**DOI:** 10.3390/genes10110938

**Published:** 2019-11-17

**Authors:** Rabiul Islam, Yefang Li, Xuexue Liu, Haile Berihulay, Adam Abied, Gebremedhin Gebreselassie, Qing Ma, Yuehui Ma

**Affiliations:** 1 Institute of Animal Science (IAS), Chinese Academy of Agricultural Sciences (CAAS), No. 2 Yuanmingyuan West Rd., Haidian, Beijing 100193, China; md.rabiul27@yahoo.com (R.I.); yefanglee1994@163.com (Y.L.); Lxx_caas@163.com (X.L.); haile.berihulay@yahoo.com (H.B.); aa.abied89@gmail.com (A.A.); gerageruggg@gmail.com (G.G.); 2 Department of Livestock Services of Ministry of Fisheries and Livestock, Farmgate, Dhaka-1215, Bangladesh; 3 Institute of Animal Science, Ningxia Academy of Agriculture and Forestry Sciences, Yinchuan 75002, Ningxia, China

**Keywords:** SNP, Chinese goat, genetic diversity, selection of signature, fecundity

## Abstract

Detection of selection footprints provides insight into the evolution process and the underlying mechanisms controlling the phenotypic diversity of traits that have been exposed to selection. Selection focused on certain characters, mapping certain genomic regions often shows a loss of genetic diversity with an increased level of homozygosity. Therefore, the runs of homozygosity (ROHs), homozygosity by descent (HBD), and effective population size (*N_e_*) are effective tools for exploring the genetic diversity, understanding the demographic history, foretelling the signature of directional selection, and improving the breeding strategies to use and conserve genetic resources. We characterized the ROH, HBD, *N_e_*_,_ and signature of selection of six Chinese goat populations using single nucleotide polymorphism (SNP) 50K Illumina beadchips. Our results show an inverse relationship between the length and frequency of ROH. A long ROH length, higher level of inbreeding, long HBD segment, and smaller *N_e_* in Guangfeng (GF) goats suggested intensive selection pressure and recent inbreeding in this breed. We identified six reproduction-related genes within the genomic regions with a high ROH frequency, of which two genes overlapped with a putative selection signature. The estimated pair-wise genetic differentiation (*F*_ST_) among the populations is 9.60% and the inter- and intra-population molecular variations are 9.68% and 89.6%, respectively, indicating low to moderate genetic differentiation. Our selection signatures analysis revealed 54 loci harboring 86 putative candidate genes, with a strong signature of selection. Further analysis showed that several candidate genes, including *MARF1*, *SYCP2*, *TMEM200C*, *SF1*, *ADCY1*, and *BMP5*, are involved in goat fecundity. We identified 11 candidate genes by using cross-population extended haplotype homozygosity (XP-EHH) estimates, of which *MARF1* and *SF1* are under strong positive selection, as they are differentiated in high and low reproduction groups according to the three approaches used. Gene ontology enrichment analysis revealed that different biological pathways could be involved in the variation of fecundity in female goats. This study provides a new insight into the ROHs patterns for maintenance of within breed diversity and suggests a role of positive selection for genetic variation influencing fecundity in Chinese goat.

## 1. Introduction

Chinese goats have extensive genetic resources and an outstretched gene pool. Natural selection and artificial selection at different intensities over time, imposed by environmental changes and animal husbandry practices, have resulted in a considerable number of desirable traits such as extensive adaptability, outstanding prolificacy, and powerful disease and cold resistance [1]. The traceable unique genetic pattern remaining in the genomic regions of an individual under selection is termed their selection signature [2]. Selection focused on certain characters, mapping certain genomic regions, often shows reduced genetic diversity and stretches of homozygosity. Detection of this selection footmark in the genomic regions can provide information regarding the underlying genetic mechanisms of specific phenotypic traits to better guide animal breeding. The genetic diversity of animals is vital for promoting their current production potential in a diverse environment, changing breeding strategies, and sustainable genetic improvement. The lack of genetic variation resulting from breeding closely related individuals often leads to the expression of genes that are detrimental to reproduction or even survival. This is why intensive selection strategies have drawn the attention of the scientific community; interest exists in preserving, characterizing, and monitoring the autozygosity of important animals for sustainable livestock production [3,4].

Inbreeding is a concerning practice in the livestock industry, considerably influencing genomic patterns, hence designing a tactful breeding scheme is essential. Practically, inbreeding represents the level of homozygosity of a population or within the genome of an individual, which leads to offspring with loss of fitness. The inbreeding coefficient (*F*) is generally used to estimate the extent of individual’s inbreeding, which is traditionally computed from pedigree information. In recent times, there is an increasing interest in estimating the inbreeding coefficient from runs of homozygosity (ROH) as it is likely to be the most powerful method in detecting inbreeding effects from among several alternative estimates. Molecular genetics has facilitated the estimation of the level of autozygosity at individual and population levels resulting from demography, natural and artificial selection, and inbreeding by checking the number of contiguous segments of the genome, referred to as runs of homozygosity (ROH) [5,6]. ROH is used to estimate the extent of identical haplotypes in the genome of an individual, transmitted from their parents [2]. A longer ROH indicates recent inbreeding, whereas a shorter ROH indicates a loss of genetic diversity resulting from the founder effect or a genetic bottleneck in a population. The selection pressure increase the homozygosity in the targeted genomic region leads to the occurrence of ROH [7]. Therefore, the genomic regions with high ROH frequency can be used to detect the association between the genes and traits of interest [8]. Characterization of the homozygosity by descent (HBD) segments associated with ROH, and estimation of the autozygosity in an individual’s genome, have become popular techniques as these measures provide insights into the recent history of a population as well as trait architecture [9]. However, until now, the extent of the ROH across the genome of various goat breeds was poorly understood.

The effective population size (*N_e_*) is the size of a hypothetical ideal population that has the same divergence of gene frequency under random genetic drift or an equal amount of inbreeding as in the real population under consideration [10]. The effective population size is an important parameter for evaluating population genetic diversity and characterizing and understanding the underlying genetic architecture of an animal genome [11]. The *N_e_* is crucial in conservation biology as it is used to estimate the rate of genetic drift and inbreeding and affects the systematic evolutionary forces such as selection, mutation, and migration [12]. The *N_e_* also provides information on population demographic processes such as migration and admixture, genetic variations, and linkage disequilibrium in a population [11,13].

Reproductive performance is characterized by fecundity, which is crucial to the goat industry. Improvement of reproductive traits, especially the increase in litter size, has attracted widespread interest because small improvements could lead to large gains in profit [14]. With the advancement of molecular genetic technologies, a series of techniques have been developed to identify evidence of selection. Numerous candidate genes and genetic loci have been identified in years using single nucleotide polymorphism (SNP) chips [15]. Due to the availability of the Illumina Goat SNP 50K BeadChip (Gene seek/Neogen, San Diego, USA), exploring selection signatures, identifying candidate genes, and genomic diversity assessments resulting from selective pressure have become the main focus of genomic studies in goats.

China is a country with rich and diverse goat genetic resources. About 58 indigenous goat breeds exist in China [16], distributed in different geographical, environmental, and agro-ecological areas, including the north pastoral region, the semi-pastoral-agricultural region, the north and south agricultural regions, and the Qinghai–Tibet plateau region [17]. In the western region of China, the Hengduan Mountains, as a natural geographical barrier, block the Qinghai–Tibet and Yunnan–Guizhou Plateaus, have the most complex river systems (Huaihe River Line), and a profoundly complex and dynamic geological history [18]. The Qinling Mountains, as a vital geographic barrier in Asia, separate the mainland of China into southern semi-tropical and northern temperate regions [19]. These mountains divide Western China into two geographic structures: Northwest China and Southwest China, and are the cause of the different climatic and agro–ecologic characteristics. Since China is a large subcontinent with diverse geographical locations, Chinese goat breeds show tremendous variations in fecundity, production traits, fiber quality, disease resistance, and heat tolerance. Different intensities of natural and artificial selection pressures force them to exhibit different phenotypic characteristics. Among Chinese goat breeds, Jinning Grey (JG), Liaoning cashmere (LN), Guangfeng (GF), Luoping Yellow (LP), Nanjiang (NJ), and Qinggeli (QG) goats are the most popular. Qinggeli and Liaoning Cashmere goats have a relatively low fecundity (~100%) compared with Jinning Grey, Nanjiang, Guangfeng, and Luoping Yellow goats (~200%). Hence, our goal was to characterize genome-wide ROH and detection of positive selection signature to provide insights into the patterns of homozygosity, population demographic history, evolutionary mechanisms, and causative genes controlling the phenotypic diversity.

## 2. Materials and Methods

### 2.1. Ethics Statement

Ethical approval for animal survival was provided by the animal ethics committee of the Institute of Animal Science, Chinese Academy of Agricultural Sciences (IAS-CAAS) with the following reference number: IASCAAS-AE-03, on 1 September 2014.

### 2.2. Sampling and Genotyping

A total of 206 goats representing six goat populations, including GF, JG, LN, LP, NJ, and QG, were selected for our study. A short introduction (breed name, sample size, fecundity rate, demography and breeding practices, average altitude and agro-ecology and feeding mode) to these goat breeds is provided in Table S1.

**Figure 1:**
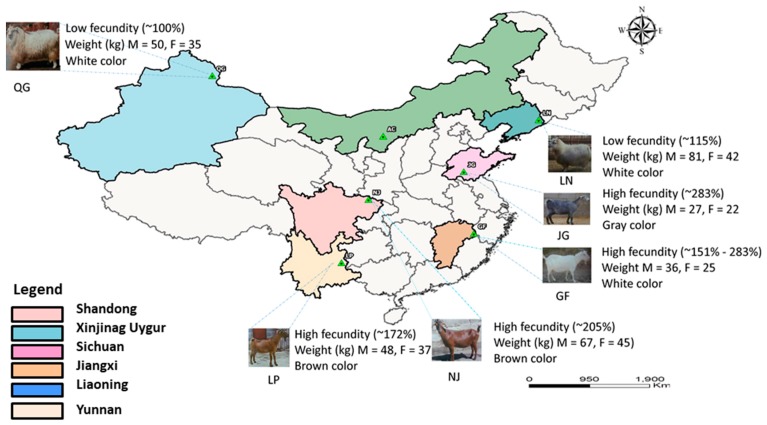
Geographic distribution and phenotypic characteristics of the six goat populations in China. M, Male; F, Female; QG, Qinggeli; LP, Luoping Yellow; NJ, Nanjiang; LN, Liaoning; JG, Jinning Grey; GF, Guangfeng.

To avoid the probability of relatedness among individuals, samples were collected from different places to represent the within-breed genetic diversity and selection of signature for the reproduction trait. The fecundity rate was obtained from the respective goat farms, where the farmers had recorded it for many years and averaged it per 1000 population for each breed. Then, the fecundity rate was calculated as many offspring can be produced by 100 female goats at one timepoint. We compared the collected fecundity rate with the literature and found similarities [17]. Sample distributions are depicted in Figure 1. Genomic DNA was extracted from blood and tissue samples using the Promega Wizard Genomic Purification Kit (Promega, Madison, USA), according to the standard protocols provided by the manufacturer. Qualified DNA was genotyped using the Illumina Goat SNP 50K Beadchip panel (GeneSeek/Neogen, San Diego, USA).

### 2.3. Data Quality Control

Quality control of the SNP data was implemented using PLINK v.1.07 [20,21]. The non-informative SNPs that were incompatible with the following criteria were removed from the panel: (1) SNP call rate greater than 95%, (2) an SNP minor allele frequency greater than 0.05, (3) an SNP with a genotyping rate greater than 95%, (4) a maximum individual missing genotype rate of more than 10%, or (5) Hardy–Weinberg equilibrium >1 × 10^−5^.

### 2.4. Detection of Runs of Homozygosity

#### 2.4.1. Distribution of ROH

ROHs were computed and characterized for each breed using detectRUNS packages in R software [20]. The following criteria were used to define ROHs: (1) a minimum number of SNPs = 15, (2) minimum length of ROH = 1 Mb, and (3) one possible heterozygote and one missing genotype were allowed for each ROH [22]. The number of ROHs per chromosome was estimated by counting the ROH number for each chromosome. The percentage of the chromosome covered by ROHs was calculated by the formula suggested by Al-Mamun et al. [23]: summing all the ROHs on a chromosome divided by the number of individuals containing ROHs on that chromosome produces the mean ROH length; the mean ROHs length of that chromosome is then divided by the respective chromosome length and multiplied by 100 to convert into a percentage. The total number, length of ROH (in Megabases), and the sum of all ROH segments (in Megabases) were calculated for each breed. We categorized the ROH lengths into six length classes (0–3, 3–5, 5–10, 10–20, 20–30, and >30 Mb) to compare the distribution of ROH length in each breed. We calculated the frequency and average ROH length for each length category. The average ROH length for each class of each breed was estimated by summing all ROH segments of each ROH length class per breed and dividing by the total number of individuals of that respective breed.

#### 2.4.2. Inbreeding Coefficient

The genomic inbreeding coefficient (*F*_ROH_) for each breed was computed using the following method [24]:

*F*_ROH_ = (L_ROH_/L_AUTO_), (1)

where L_ROH_ is the total length of ROH of each individual in the genome and L_AUTO_ is the length of the autosomal genome of the goat (set to 2399.4 Mb [22]). To verify the accuracy of *F*_ROH_, we also calculated the inbreeding coefficient based on the difference between the expected and observed numbers of homozygous genotypes (*F*_HOM_) with the command–het using PLINK v.1.07. The correlation between *F*_ROH_ and *F*_HOM_ was calculated across the breeds.

#### 2.4.3. Detection of Common ROHs

To identify the genomic regions with a high ROH frequency, the percentage of occurrences of an SNP in an ROH was calculated by counting the number of times the SNP was detected in an ROH across the population. The SNPs showing a percentage higher than 45% were selected as genomic regions with a high frequency of ROHs for further analysis. We used the caprine reference genome annotation file from the NCBI website to annotate the genes identified at particular genome coordinates for all the selected regions [25].

### 2.5. Homozygosity by Descent (HBD)

A hidden Markov model (HMM)-based approach was implemented in R CRAN: (https://CRAN.Rproject.org/package=RZooRoH) to scan the individual genome for the HBD segments, as described in Solé and Gori [26] and Bertrand and Kadri [9]. A MixKR model was applicable, where nine HBD states with respective rates (*R_k_*) of (2^1^, 2^2^, 2^3^, …, 2^9^) and one non-HBD with an *R_k_* rate of 2^9^ were used. The number of classes, K, was fixed for each run. Only one non-HBD class and *K*—1 HBD class for a total of K = 10 class were considered. The predefined rate of *K*—1 HBD classes always ranged 2 to 2*^K^*^—1^, whereas the rate of the non-HBD class was fixed as the most ancient class. Each K has its own rate parameter, *R_K_*, which indicates the lengths of the segments for its respective class. The length of HBD classes is exponentially distributed with rate *R_k_*, which is double the number of generations to the common ancestor of the respective class, and this is called a K.R model [26]. The length of the HBD segment is 1/R Morgans, indicating high rates associated with shorter segments. To estimate the inbreeding coefficient, we considered the ancestors with an *R_k_* rate higher than threshold *T* as unrelated. The corresponding genomic inbreeding coefficient (*F_G−T_*) was then estimated, with *R_K_ ≤ T* averaged over the whole genome.

### 2.6. Effective Population Size (N_e_)

Effective population size (*N_e_*) was calculated for each breed separately using SNeP v1.1 [27]. *N_e_* estimates at different time points are based on linkage disequilibrium, using the formula proposed by Sved [28]:

(2) $$E(r^{2})\;=\;\frac{1}{1+4N_{e}c}\;,$$

where *N_e_* is the effective population size, *c* is the genetic distance in Morgans, and *E*(r^2^) is the expected average coefficient of determination (*r*^2^) value for distance *c*. Each time point corresponding to a genetic distance was calculated as: *T* = $\frac{1}{2c}$. Estimated effective population sizes (*N_e_*) against their past generations were plotted in R over the last 100 and 1000 generations.

### 2.7. Population Differentiation, Analysis of Molecular Variance, and Structure

Reynolds’s genetic distance and pairwise difference were computed using the integrated software for a different hierarchical level of population genetic data analysis, using ARLEQUIN v.3.5.2 [29] with 100 permutations and a significance level of 0.05. The analyses of molecular variance (AMOVA) to test the partition of genetic diversity were performed using ARLEQUIN v.3.5.2.

#### 2.7.1. Principal Component Analysis

To investigate the genetic relationship between individuals, we used the principal component analysis (PCA). A pruned set of SNPs that were in approximate linkage equilibrium with each other were used to avert clustering [30]. The PLINK indep-pairwise option (indep-pairwise 50, 10, 0.1), where the command considers each window of 50 SNPs, was used to find the pruned set of SNPs [20]. The criterion of a pairwise linkage disequilibrium (*r*^2^) value of <0.1 was used to obtain the SNP panel that approximates linkage equilibrium. In this study, PCA was performed using PLINK software (parameter: pca) and the output was visualized using R software.

#### 2.7.2. Neighbor-Joining Tree

To further confirm the phylogenetic relationships among the breeds, we constructed a neighbor-joining (NJ) tree using MEGA v.5.0 [31]. We calculated the genetic distance matrix using PLINK v.1.09 [20,21] (parameter: distance-matrix) and then, based on this distance matrix, we constructed the Neighbor-joining tree using MEGA v.5.0.

#### 2.7.3. Admixture Analysis

To examine the pattern of genetic variation among the breeds, a model-based clustering approach was applied using the ADMIXTURE program v.1.2 [32]. The ancestral source K refers to the number of populations used in this dataset. A five-fold cross validation (CV) error for each K was used to select the best K.

### 2.8. Selective Sweep Analysis

We used the population differentiation index (*F*_ST_), nucleotide diversity (π), and cross-population extended haplotype homozygosity (XP-EHH) approach to identify the genomic regions that appear to be the target of selection. We categorized the goat breeds into high and low groups according to their reproductive performance, and compared the high fecundity (~200%) goat breeds group (JG, GF, NJ, and LP) with the low fecundity (~100%) goat breeds (LN and QG) to explore positive selection signatures.

#### 2.8.1. Population Differentiation Index (*F*_ST_) Approach

The population differentiation index *F*_ST_ is an important indicator for testing the footprint of positive selection and elicit whether genetic differentiation exists between populations. The unbiased metric of pairwise *F*_ST_ values were computed, as described by Weir et al. [33]. We then transformed *Z* into *F*_ST_ values [34].

#### 2.8.2. Nucleotide Diversity (π)

Another popular approach to calculate the degree of polymorphism within a population is nucleotide diversity (π). We calculated the site nucleotide diversity (π) as the proportion of pairwise differences between two populations, (π (low fecundity goat)/π (high fecundity goat)) to investigate the genetic variation between the groups.

#### 2.8.3. Estimation of Cross-population extended haplotype homozygosity (XP-EHH)

The XP-EHH estimate is often used to detect a positive selection event, mainly to compare the haplotype homozygosity (EHH) and integrated haplotype score (iHS) between two populations. The basic principle of XP-EHH estimation involves scanning the distinct SNPs between populations that are homozygous for one and polymorphic for others through the comparison of the EHH score of two populations. A positive XP-EHH value indicates selection occurs in the test population; a negative value indicates selection in the control population. The calculation formula is

(3) $$XP-EHH=ln\left(\frac{I_{A}}{I_{B}}\right),$$

where *I_A_* is the integrated value of the test population EHH and *I_B_* is the integrated value of the reference population EHH. The codes from the following website were used to calculate the XP-EHH: http://hgdp.uchicago.edu/software/xpehh.tar. The calculated raw XP-EHH statistics were then standardized to a distribution with zero mean and unit variance.

The regions showing extremely high *F*_ST_ values and elevated nucleotide diversity (top 1% of both) were considered to be potentially selected candidate outliers under selection. We also scanned the top 0.1% of XP-EHH estimates to improve the confidence in the selected outliers under selection.

#### 2.8.4. Gene Annotation and Functional Enrichment Analysis

Based on the above findings, the cross top 1% values of *F*_ST_ and π and 0.1% of XP-EHH were considered as selective signals and were extended up to 100 kb upstream and downstream to cover the candidate region. To annotate the candidate region, we downloaded the caprine gene from Ensemble (http://www.ensemble.org/) and annotated it using R v.3.5.1. The overlapping genes retrieved from the two analytical approaches were used for enrichment analysis. Gene ontology (GO) and Kyoto Encyclopedia of Genes and Genomes (KEGG) pathway analysis of the target genes were performed using a web-based toolset: g: profiler (https://biit.cs.ut.ee/gprofiler/gost) and KOBAS v.3.0 (http://kobas.cbi.pku.edu.cn/anno_iden.php), respectively. As only limited genes are annotated in the goat genomes, we used the human genome as the background for enrichment analysis.

## 3. Results

### 3.1. Sample and SNP Filtration

From the 206 individuals tested, only two individuals (one from JG and another from LN) were removed from further analysis due to their low genotyping rate (Maximum individual missingness rate >0.1). The total genotyping rate in the remaining individuals was 0.98147. A total of 1338 SNPs with a call rate <0.95, 2296 SNPs with a minor allele frequency <0.05, and 1814 SNPs with a Hardy–Weinberg equilibrium (*p* < 1 × 10^−5^) were excluded from the panel (Table 1). After the quality filtration and exclusion of SNPs from the sex chromosome, 45,311 SNPs from 204 individuals were used for downstream analyses.

### 3.2. ROHs

#### 3.2.1. ROH Patterns

**Figure 2:**
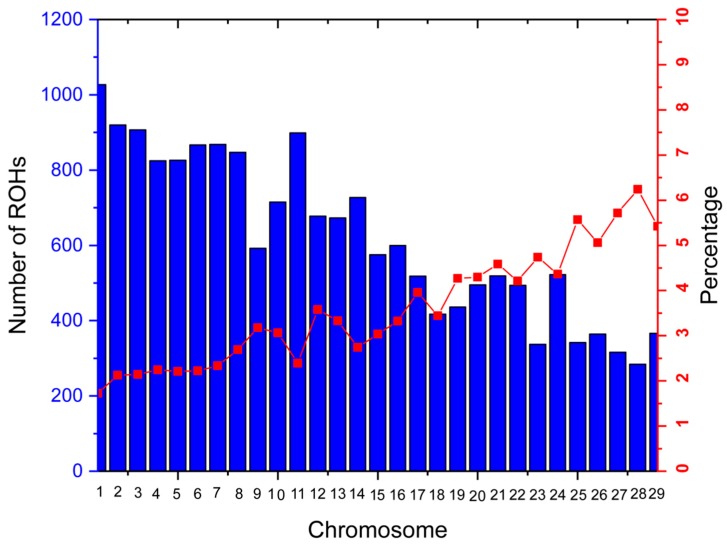
Number of runs of homozygosity (ROHs) per chromosome identified in 204 goat breeds that had at least one ROH (bars), and the percentage of each chromosome covered by ROHs (lines).

ROHs, representing the level of genomic autozygosity, are continuous homozygous segments at the individual and population levels that can be used as a measurement of inbreeding; more in-depth ROHs are the result of demography, natural and artificial selection, and inbreeding [6,35]. According to the parameters set, 18,066 ROHs were found in total, with a mean of 88.55 per individual, ranging from 11 (QG) to 244 (GF). LN presented the largest number of ROHs (*n* = 7425), followed by GF (*n* = 3415). GF displayed the longest ROHs on average (mean = 3.29 ± 0.001 Mb) and the shortest average ROH length was found in LP (1.95 ± 0.001 Mb). Among all the ROHs, the longest ROH was detected on chromosome 20 of the LP goat breed (67.54 Mb), consisting of 1304 SNPs (Table 2). A total of 812 ROHs were longer than 10 Mb (225 for GF, 78 for JG, 375 for LN, 80 for LP, 33 for NJ, and 21 for QG). Chromosome 1 exhibited the maximum number of ROHs (*n* = 1027), followed by chromosome 2 (*n* = 920) (Figure 2). The total number of ROHs per chromosome tended to decrease with decreasing chromosome length. The highest and lowest percentages of ROHs per chromosome were calculated for chromosome 28 (6.23%) and chromosome 1 (1.72%), respectively (Figure 2).

**Figure 3:**
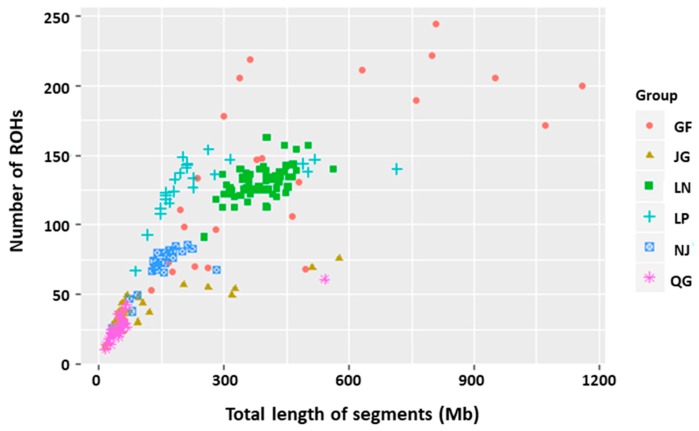
Total genomic length (Megabases) covered by ROH per individual (*x* axis) and total number of ROH per individual (*y* axis).

Figure 3 shows the relationship between the number of ROHs and the total genomic length covered by ROHs per individual, which varies considerably among breeds. The QG breed presented with a large number of short ROHs relative to the other breeds, and the GF goat displayed some extreme individuals with ROH coverage of more than 600 Mb (Figure 3).

**Figure 4:**
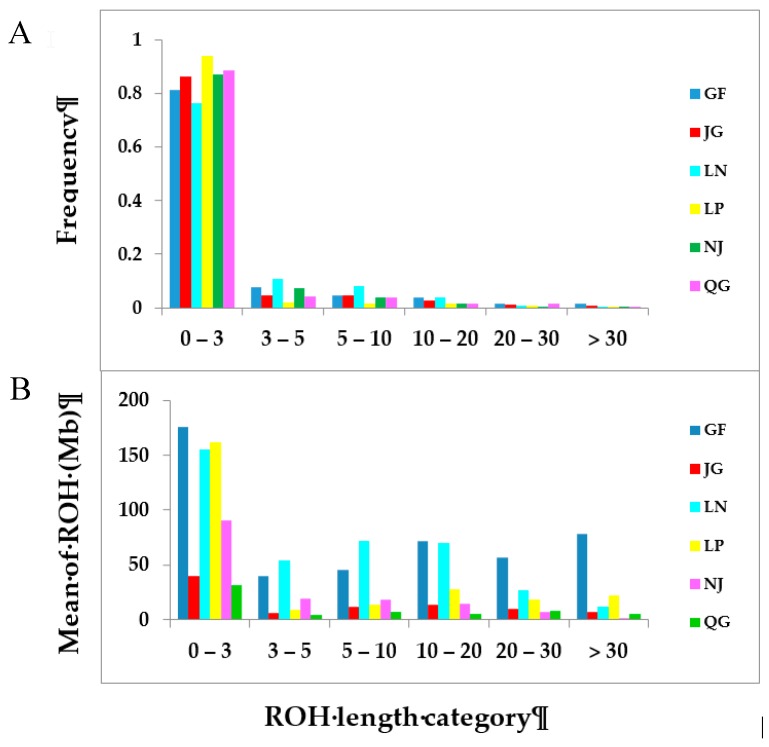
(**A**) Frequency of ROHs in different length categories in six goat populations. (**B**) The average sum of runs of homozygosity (ROH) of each breed in different ROH length classes.

The frequencies of ROH numbers in different length classes are illustrated in Figure 4A. Our results show an inverse relationship between ROH length and frequency. The frequencies of shorter ROHs (0–3) predominated; the number of these ROHs accounted for 82.72% of the total number of ROHs. However, the frequency of ROHs in this major class varied between breeds. The majority, more than 90% of the ROHs in this class, were obtained from LP breeds, whereas the percentage was at least 80% for GF, JG, NJ, and QG, and less than 80% for the LN breed. GF and LN had the highest frequencies of ROHs among all breeds in the length category greater than three. Figure 4B shows the distribution of ROH within breeds. Among the six ROH categories under consideration, the short ROHs (<3) are most prevalent across the populations, wherein the average ROH mean spanned from 31 (QG) to 175 (GF). The longest ROH category (>30) was the rarest, wherein the GF breed displayed the highest mean ROH compared with the other breeds.

**Figure 5:**
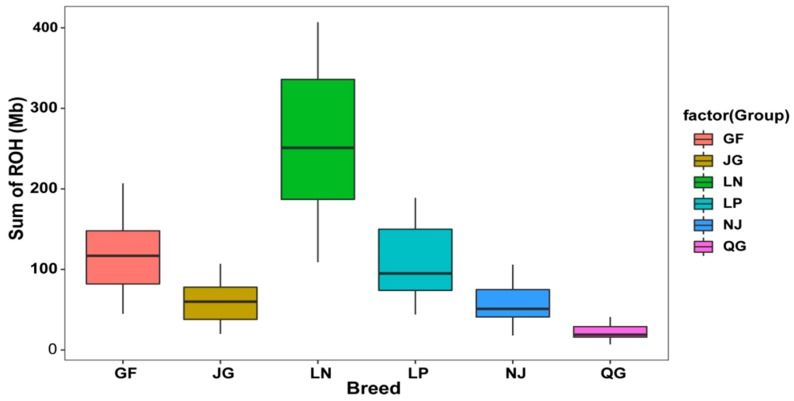
The sum of ROH length (Mb) per individual genome in each breed.

To investigate the ROH content in each breed, we also calculated the sum of all ROH lengths for each individual within the breeds and found that the LN had more ROHs than others (Figure 5). Individuals of the LN breed generally exhibited the highest average sum of ROH content (256.03 Mb) on their genome, followed by GF (117.75 Mb) and LP (106.75 Mb) (Figure 5, Table S2). In contrast, the QG individuals displayed the lowest average sum of ROH (22.20 Mb).

#### 3.2.2. Inbreeding Coefficient of Runs of Homozygosity (*F*_ROH_)

**Figure 6:**
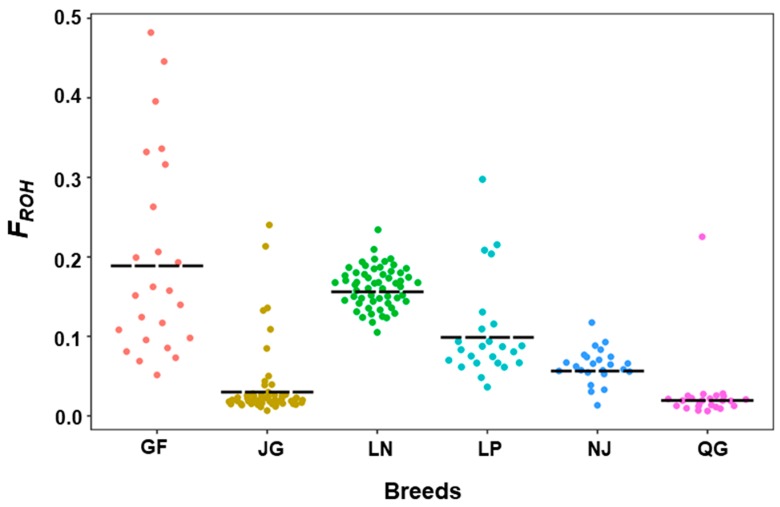
Distribution of the runs of homozygosity inbreeding coefficient (*F_ROH_*) within each breed.

The mean inbreeding coefficient estimates, with their range in variation and distribution in the studied goat breeds, are reported in Table 2 and Figure 6. The mean *F*_ROH_ for all breeds ranged from 0.026 (QG) to 0.19 (GF) (Table 2). The GF goat is the most inbred goat, followed by LN (0.162) and LP (0.105). At the individual level, the individuals of the GF breed displayed the highest *F*_ROH_ (0.48), including some individuals with extreme values compared with other breeds.

The *F*_HOM_ results indicated positive values only for the GF and LP breeds. The *F*_ROH_ was higher in GF in both the short- and long-length classes, whereas LN showed a higher *F*_ROH_ in the medium-length class (Table 3). The correlations between *F*_ROH_ and *F*_HOM_ were found to be highest for QG (0.99), followed by NJ (0.98) and GF (0.97) (Table 2). The *F*_ROH_ was higher in GF in most of the ROH length classes, with a higher mean *F*ROH across the classes (Table 3).

#### 3.2.3. Genomic Regions with a High ROH Frequency

**Figure 7:**
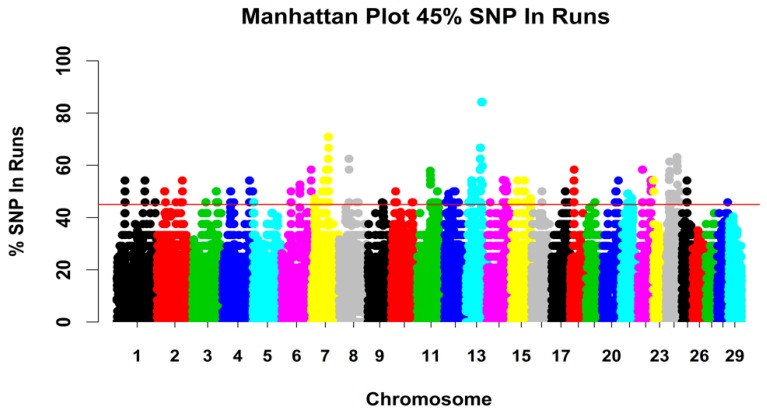
Manhattan plot of the occurrences (%) of an SNP in ROHs across the population. A dot represents an SNP.

The most common genomic regions associated with ROHs in the six goat populations were detected, and the percentage of SNPs in ROHs were assessed by calculating the frequency of SNPs occurring in those ROHs across individuals. The output was plotted against the position of the SNP along the chromosome (Figure 7). A total of 97 genomic regions were identified (Table S3). Here, we focused on the selected regions and investigated if the identified regions coincided with the regions harboring the genes involved in reproduction. We identified six genes in the selected ROH regions that are associated with reproduction. We found that the ROHs on chromosomes 25 and 13 overlapped with regions detected by the selection signature, which spanned *MARF1* and *SYCP2*, respectively. Another two regions on chromosomes 4 and 24 were near to the region detected by the selection signature, which contain *ADCY1* and *TMEM200C*, respectively; these genes are also associated with reproductive processes.

### 3.3. Homozygosity by Descent (HBD) Classes

The inheritance of two copies of the same chromosomal segments in an individual’s genome from an ancestor tracing back to different periods is referred to as homozygosity by descent (HBD) segments. The variable sizes of these segments are associated with the different ancestors that can be traced back to different generations in the past. The rate of the HBD class is inversely associated with the size of the segments. Long HBD segments correspond to recent common ancestors, whereas short segments indicate autozygosity inherited from ancient common ancestors.

**Figure 8:**
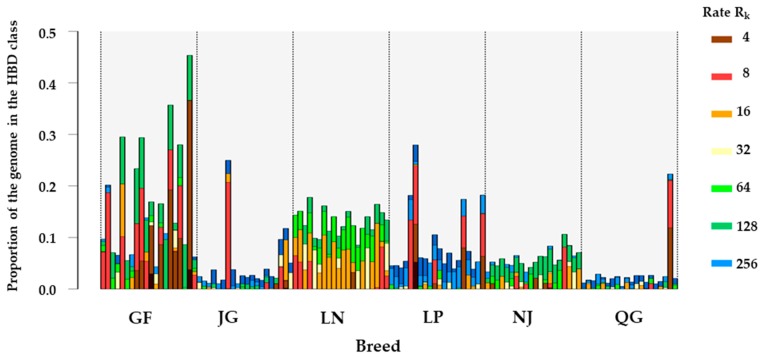
Partitioning of the genome in different homozygosity by descent (HBD) classes in six goat populations. The results are plotted for 20 randomly selected individuals per population. The height of each bar represents the proportion of the genome associated with the HBD class of the corresponding color.

Figure 8 presents the partitioning of individual genomes in seven different HBD classes. A difference was observed in terms of partitioning. The individuals from JG and QG showed a limited amount of autozygosity associated with small and ancient HBD segments (indicated in blue). The GF breed displayed more variation in autozygosity and length of HBD segments. The most inbred GF population exhibited an autozygosity level of more than 0.4, mainly associated with a distant ancestor, with indications that the ancestors contributed traces back 128 generations. Some individuals of GF exhibited HBD segments from recent common ancestors. LN individuals showed a higher level of autozygosity compare with the LP and NJ breeds, but this is associated with more distant ancestors (with the HBD classes with ratings of 16 to 128).

**Figure 9:**
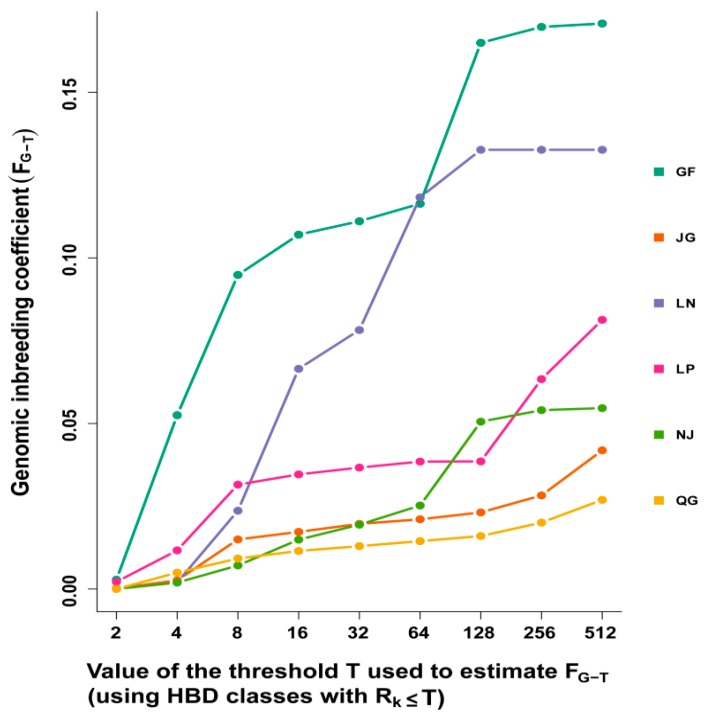
Value of the threshold *T* used to estimate *F_G–T_* (using HBD classes with *R_K_ ≤ T*).

The average inbreeding coefficient was estimated using the HBD class. GF goats displayed the highest average inbreeding coefficient, followed by the LN breed. An increasing trend of inbreeding coefficient with ancient classes was observed across the breeds, whereas the GF goats showed a rapid increase in the inbreeding coefficient from 0.00 for an estimate based on the first class (R_K_ = 2) to 0.09 with R_K_ ≤ 8, and then increasing marginally up to R_K_ ≤ 64. The highest value was observed at R_K_ = 512 (0.169) (Figure 9). A similar pattern for the inbreeding coefficient was found for LN: rapidly increasing up to R_K_ ≤ 64, and the highest inbreeding coefficient was found at R_K_ ≤ 128 (0.134).

### 3.4. Effective Population Size (N_e_)

**Figure 10:**
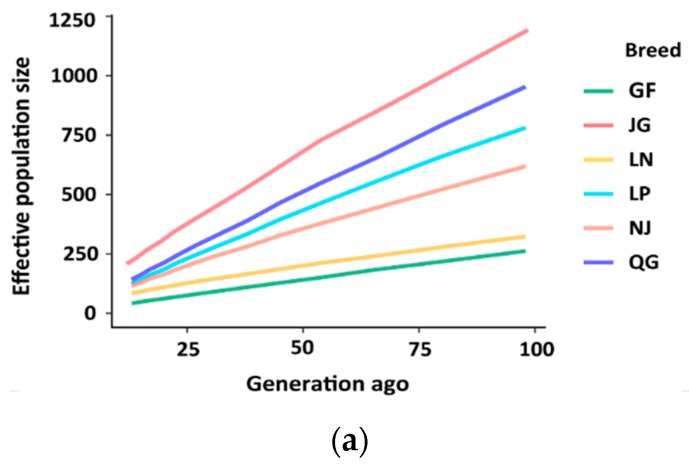

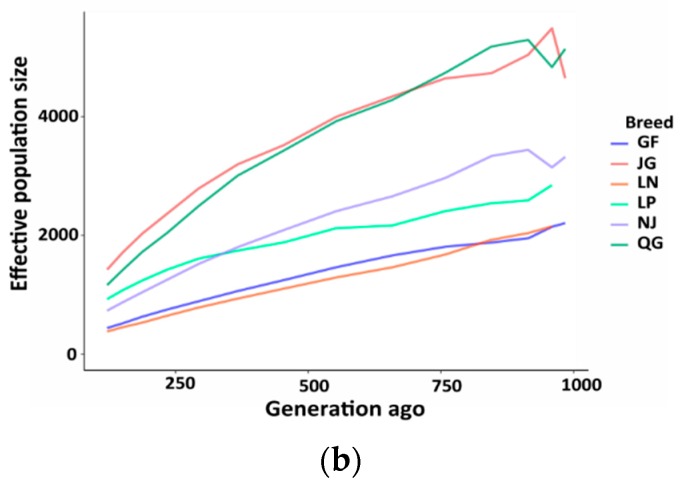
Estimated effective population sizes (*N_e_*) in six Chinese goat populations. (**a**) *N_e_* in the past 100 generations. (**b**) *N_e_* over the past 1000 generations.

Ancestral and recent effective population sizes (*N_e_*) for six goat populations are presented in Figure 10. Estimated *N_e_* showed a downward trend with the increase in generations across the populations. The most rapidly declining recent *N_e_* was found in the GF and LN breeds, whereas JG and QG showed a slowly declining trend in *N_e_* (Figure 10b).

### 3.5. Population Differentiation, AMOVA, and Structure

Population genetic differentiation was evaluated by calculating Reynolds’ genetic distance and pairwise differences. Our result showed that the Reynolds’ genetic distance (D_R_) values vary from 0.02116 (NJ–QG) to 0.20055 (LN–LP), whereas the highest and lowest pairwise differences were found for LN–LP (0.18172) and NJ–QG (0.02099), respectively (Table 4).

Analysis of molecular variance (AMOVA) illustrated that intra-population variation accounted for 89.61% of the total variation, with 9.68% (*p* < 0.001) of the variation being inter-population (Table S4).

### 3.6. Detection of Signature of Selection

#### 3.6.1. Distinct Population Structure Pattern

**Figure 11:**
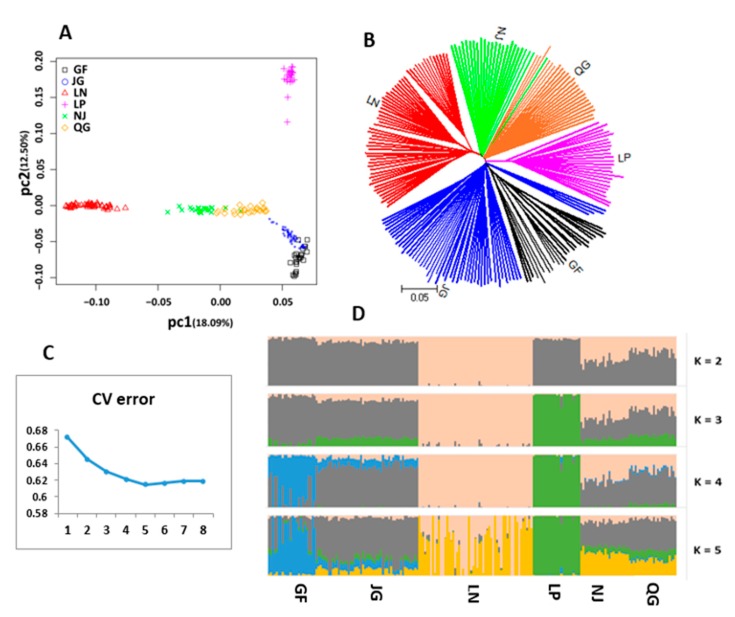
Phylogenetic relationships of six goat populations. (**A**) Principal component analysis of 204 individuals. (**B**) Neighbor-joining phylogenetic tree of goat breeds. (**C**) Cross-validation of six goat populations. (**D**) Genome-wide admixtures inferred by ADMIXTURE software.

The PCA results showed that the first principal component (PC1) accounted for 18.09% of the genetic variation, resulting in the clear segregation of LN and a clear separation of the LP breed in the positive direction of the second principal component (PC2), which explained 12.50% of the total variance (Figure 11A). The NJ breed is clustered together with QG and a degree of overlap was detected between JG and GF, which is in accordance with their geographic distribution in China (Figure 1). This finding is consistent with the result of the NJ tree, wherein the LN and LP breeds formed genetically distinct groups (Figure 11B). The NJ and QG goat breeds are clustered together and the degree of differentiation was low between JG and GF in the NJ tree analysis (Figure 11B). To further verify the PCA results, we performed population admixture analysis, where the least amount of cross-validation error occurred when K = 5 (Figure 11C), which indicates that K = 5 was the optimal modeling choice. Our admixture result showed that the LN and LP goat breeds displayed a separate group when K = 3. However, the QG admixed with NJ and JG, and GF goat breeds are mixed with each other (Figure 11D) when K = 4 or 5. The results of the admixture were consistent with the results of PCA and the NJ tree.

#### 3.6.2. Genome-Wide Selective Sweep Analysis in High-Fecundity Goat Breeds (High vs. Low)

**Figure 12:**
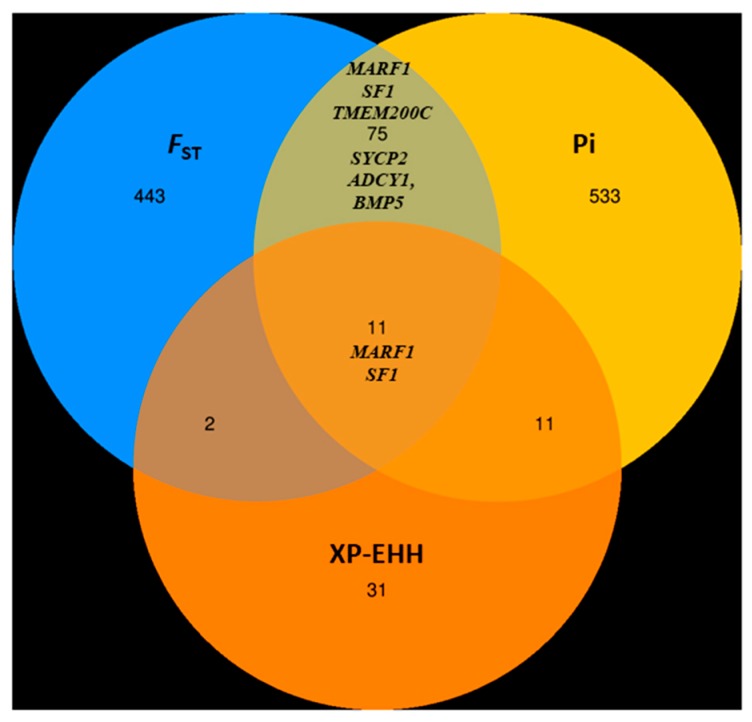
Venn diagram showing the overlapping genes in the top 1% of population differentiation index *(F*_ST_), nucleotide diversity (π) and 0.1% of Cross-population extended haplotype homozygosity (XP-EHH).

**Figure 13:**
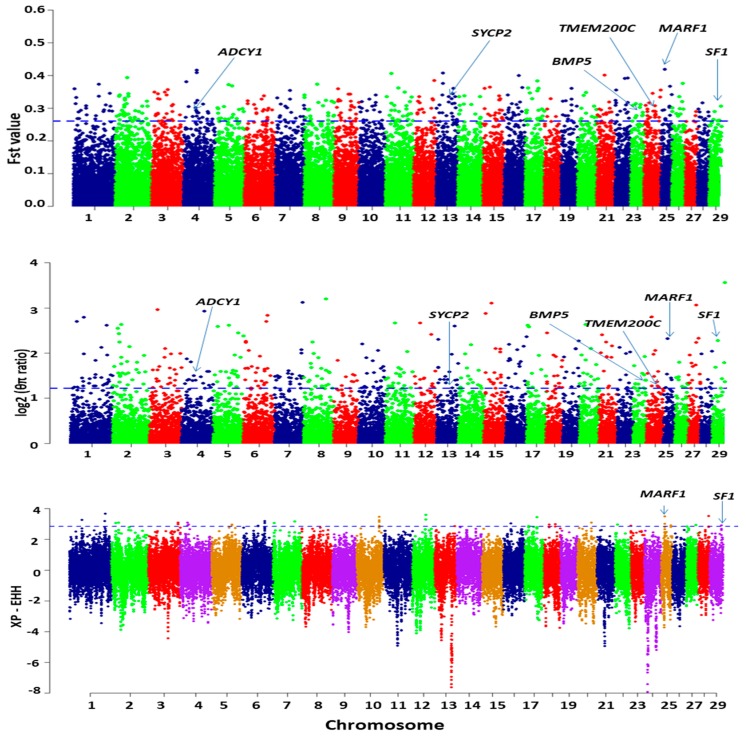
Selection signature detection in the high fecundity group of goat populations. Dotted lines indicate the top 1% values of *F*_ST,_ and π, and 0.1% values of XP-EHH.

The regions falling within the upper 1% of the empirical distribution of *F*_ST_ (*F*_ST_ > 0.261025) and nucleotide diversity (π) were scanned as candidate regions under selection. Based on the reference genome annotation, a gene that overlapped with an outlier of the top 1% of both the *F*_ST_ and π estimates was deemed to be candidate gene under positive selection. We identified 460 candidate loci, of which a subset of 54 loci harbored 86 putative candidate genes with the signature of positive selection (Table S5). To improve confidence, we also scanned the top 0.1% of XP-EHH estimations. We found two candidate genes were highly differentiated between the high and low-reproduction groups according to three approaches used (Figure 12, Figure 13) that might have experienced strong positive selection. Based on their biological functions, associated pathways, and information from published studies, several genes were found possibly responsible for the reproduction trait in goats and are thus annotated in the Manhattan plot of *F*_ST_, log_2_ (θπ ratio) and XP-EHH (Figure 13). Table 5 shows the potential candidate genes with their loci.

#### 3.6.3. GO Annotation and KEGG Pathway of the Target Genes

**Figure 14:**
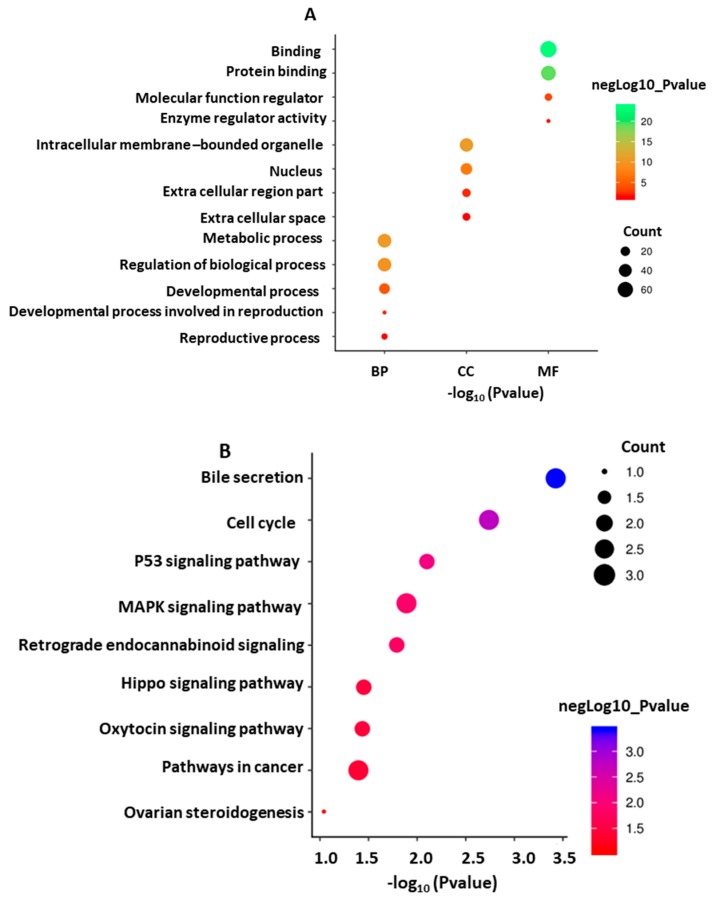
The bubble chart of (**A**) gene ontology (GO) terms and (**B**) Kyoto Encyclopedia of Genes and Genomes (KEGG) pathways of the selected genes.

Functional enrichment of the target genes revealed that the genes were significantly enriched in 20 GO terms in molecular functions, 103 terms in biological processes, and 49 terms in cellular components (Table S6). The most significant GO terms were found in metabolic processes, regulation of biological processes, developmental processes, and reproductive processes in BP, binding (GO: 0005488) in molecular functions, and intracellular organelles (GO: 0043229) in cellular components (Figure 14A).

A total of 25 KEGG pathways were found to be significantly enriched at the threshold (*p* < 0.05), although 93 pathways were involved with these genes. The most enriched KEGG pathways included “bile secretion”, “cell cycle”, “p53 signaling pathway”, “Mitogen-activated protein kinase (MAPK) signaling pathway”, “retrograde endocannabinoid signaling”, “hippo signaling pathway”, “oxytocin signaling pathway”, “pathways in cancer” and “ovarian steroidogenesis” (Figure 14B, Table S7).

## 4. Discussion

The detection of selection footprints in the genomic region has the potential to be used to identify genes and mutations associated with economically important phenotypic traits of livestock species. As the level of genetic diversity represents the raw materials for breed improvement, elucidating the genetic diversity of a population can provide insights for improving breeding strategies. ROH, HBD, and *N_e_* are useful tools for exploring genetic diversity, providing information about population demographics evolution over time, and predicting underlying genome architecture. The developed genome-wide goat SNP arrays have become the marker of choice for investigating underlying genetic diversity, inferring population demography, and mapping genomic regions subject to selection [36]. In this study, we examined the genome-wide runs of homozygosity, effective population size, and signature of positive selection in six Chinese goat populations using goat SNP 50 K chips.

Our findings showed that the ROHs are frequent across the populations. The distribution of ROHs per chromosome displayed a specific pattern: the greatest number was found in the first three chromosomes, the ROH number tended to decrease with decreasing chromosome length, and the smallest number was found on chromosome 28, consisting of 284 segments. Our results are consistent with those reported for Valle del Belice sheep [37]. The percentage of coverage per chromosome was quite different in the different populations, which suggests it may be breed-specific.

The higher sum of ROH length across the GF goats indicated their lower level of genetic diversity. In general, all breeds showed their majority of average ROHs in the 0–3 Mb length class, which is in agreement with the results obtained for Spanish goats [34]. A different ROH distribution pattern was noted for GF goats, which displayed a high mean ROH in the long length category (>30 Mb), which is indicative of demographic decline and recent inbreeding [38]. Thus, the accumulation of long ROHs in the genome of the GF breed enables them to carry deleterious mutations in homozygous form [39]. The highest value of *F*_ROH_ in GF, suggesting high inbreeding in this goat breed, is the signature of the extensive use of few bucks within herds. Consequently, widespread mating between relatives occurred, which might have contributed to the high proportion of fixed alleles, resulting in low genetic diversity in the GF population. Attention should be paid to this goat breed to prevent a loss of goat genetic resources. A large contribution to autozygosity was observed in GF goats in the HBD class, tracing back to different generations, which indicates a reduced effective population size, possibly due to a bottleneck or the founder effect. A similar trend in autozygosity was observed for sheep [40]. The limited amount of autozygosity in JG and QG goat populations suggested a large *N_e_* in the past. We found a declining trend in *N_e_* from 1000 to 100 generations ago across the studied populations. This decreasing trend in *N_e_* might be associated with human migration with goats that subsequently led to breed formation. The most rapid decline in *N_e_* was observed over the last 100 generations in all breeds, suggesting that significant bottlenecks occurred at the time of domestication, breed formation, and selection within these breeds [41,42]. The elevated *N_e_* estimates in JG, LP, NJ, and QG in all generations compared with GF and LN might be due to population admixture within these breeds. Brito and Jafarikia [43] estimated the *N_e_* in Australian and Canadian goats and found a similar trend of declining *N_e_* over the past generations, and an accelerated decline in *N_e_* over the past 100 generations. Comparatively, a lower *N_e_* was found in GF and LN, with estimated values were 42 and 85, respectively, 13 generations ago, which could be due to intensive selection pressure or artificial insemination used to develop these breeds. *N_e_* values for Argentinian, French, and South African goat breeds were reported to be 57, 67, and 93, respectively, at 10 generations ago [44], which is consistent with our findings. The *N_e_* slope for GF and LN in Figure 10b indicates constantly decreasing population sizes for these breeds, suggesting that action is needed to maintain a sufficiently large *N_e_*.

All the pairwise *F*_ST_ values between populations were statistically significant (*p* < 0.05). The pair-wise *F*_ST_ values ranged from 0.02094 (NJ–QG) to 0.18172 (LN–LP), revealing the least differentiation between NJ and QG, whereas the highest differentiation was between LN and LP. In terms of genetic distance, NJ and QG are most closely related (D_R_ = 0.02116), whereas LN and LP appear most distinct (D_R_ = 0.20055), which is possibly due to shared common ancestry between NJ and QG and the geographical isolation of LP goats from other breeds. The LN is the most popular goat breed due to its cashmere trait and has experienced strong selection pressure. The LP breed is traditionally reared in the Yunnan province, in the southwest part of China. The Hengduan Mountains in between the Qinghai-Tibet Plateau and Yunnan-Guizhou Plateau have restricted livestock gene flow between Southwestern and Northern China. The results are similar to those reported for Ethiopian sheep breeds [45]. The AMOVA revealed that 89.61% of the genetic variation was within populations and 9.68% (*p* < 0.001) was between populations. This is a little higher than the within-population variation and slightly lower than between-population variation (87.86% and 11.86%) observed for South African, French, and Argentinian goat populations [44], and similar (89.83% and 7.49%, respectively) to South African and Italian goat breeds in terms of within breed variation, as reported by Nicoloso and Bomba [13].

### 4.1. Candidate Genes in the ROH Regions

In this paper, we do not discuss all the genomic regions associated with a high ROH frequency; we focused on some selected regions that showed associations with the reproductive traits in goats. We identified eight genes reported to be associated with the reproductive traits of goats (Table 6) of which two genes (*MARF1* and *SYCP2*) overlapped with the genes identified by the selection signature. The *GDF9* gene was identified on chromosome 7 of GF goats, which plays a crucial role in early folliculogenesis in female mammals [46]. *GDF7*, which is the member of the *BMP* family and is required for seminal vesicle development, was detected on chromosome 11 [47]. *INHA*, located on chromosome 2, was reported as a candidate gene for litter size in goats [48]. We identified another gene, *MTHFSD*, on chromosome 18 of LP goats, which was reported to be involved in the variation of litter size [49]. *MARF1*, which is essential for the development of competent oocytes for successful fertilization, was detected on chromosome 25 [50]. On chromosome 13 of GF goats, we identified the *SYCP2* gene, which is required for maintaining normal male and female fertility [51]. Another two genes (*TMEM200C* and *ADCY1*), which we identified by selection signature detection, were also close to the ROH region and involved in reproductive functions.

### 4.2. Signature of Selection

For selection signature scanning, the six goat populations were divided into two groups based on their genetic pattern and fecundity rate collected from the records and literature. Phylogenic analyses supported the relatedness among the breeds of the high fecundity group and clearly separated the low from the high group. A strong genetic relationship was observed between NJ and QG as they are grouped closely in the neighbor-joining tree, indicating a high degree of gene flow due to being near to each other in geographic location. The tree also showed that the GF and JG breeds form a group in the same clade, suggesting that these goat breeds share common ancestry. This result is supported by the PCA, where JG and GF are clustered together. Population admixture analysis generated some signals of admixture and underlying genetic relationships among the populations. The high fecundity group included JG, GF, LP, and NJ (~200%), which have a distinct pattern, although the NJ goat was admixed with QG goat in the admixture result. The low fecundity group contained LN and QG (~100%), and the LN goat was separated from the others in all the phylogenetic analyses.

Selective sweep based on population differentiation (*F*_ST_) and nucleotide diversity (π) for the high and low reproduction groups of goat breeds was detected. To minimize the false positive, we selected only the overlapping genes on the top 1% of *F*_ST_ and Pi approaches. To find the high-quality candidate genes, we also scanned 0.1% empirical distribution of XP-EHH estimates and selected the overlapping genes under these three approaches as strong candidate genes. Candidate genes associated with reproduction were identified in different genomic loci under selective sweep. Locus 13,838,569 of chromosome 25 had the highest *F*_ST_ value (*F*_ST_ = 0.419, Z*F*_ST_ = 6.129) and an elevated π ratio and XP-EHH (θπ ratio = 5.00, XP-EHH = 3.486; Table 5). Based on the genome annotation, this locus encompasses *MARF1* (*Meiosis arrest female 1*), which is required for the process of controlling meiosis and the development of healthy offspring. Accurate completion of the meiotic process, cytoplasmic maturational events, and genomic integrity is essential for the production of oocytes suitable for fertilization and embryogenesis. Genetic control of these events is vital for successful reproduction. A previous study showed that *MARF1* is essential for the development of fertilization competent oocyte, and mutations lead to female infertility in mammals [50]. *TMEM200C* (*Transmembrane Protein 200C*) was identified at locus 39,657,370 in chromosome 24 (*F*_ST_ = 0.306, Z*F*_ST_ = 4.281, θπ ratio = 2.409). *TMEM200C* is commonly involved in the communication of a cell with its external environment. A Genome-Wide Association Studies reported that an SNP in the proximity of the *TMEM200C* gene is significantly associated with the twinning rate in Maremmana cattle [52].The candidate gene *SF1* (*Steroidogenic factor-1*), located at locus 43,352,393 on chromosome 29 (*F*_ST_ = 0.305, Z*F*_ST_ = 4.262, θπ ratio = 3.450, XP-EHH = 2.903), is required for the formation of gonads [53] and to maintain normal reproduction [54]. The distant locus 55,939,729 of chromosome 13 showed high differentiation (*F*_ST_ = 0.329, Z*F*_ST_ = 4.647, θπ ratio = 2.346) and was detected as a putative selection signal. This locus contains *SYCP2* (*Synaptonemal Complex Protein 2*), which is a proteinaceous structure required for the normal meiotic fusion of oocytes and spermatocyte formation and to maintain normal male and female fertility. A study suggested that *SYCP2L*, which is the paralog of *SYCP2*, plays a significant role in the survival of oocytes and regulates reproductive aging in females [51]. We detected *ADCY1* (*Adenylate cyclase 1*), which showed evidence for positive selection for reproduction and is located at locus 43,874,883 on chromosome 4 (*F*_ST_ = 0.301, Z*F*_ST_ = 4.193, θπ ratio = 2.829). The candidate gene *ADCY1* encodes adenylate cyclase 1, which is involved in the formation of cyclic adenosine monophosphate (cAMP) cyclizing AMP, which subsequently partakes in oocyte meiotic arrest and suppresses its resumption [55]. *BMP5* (*Bone Morphogenetic Protein 5)* was identified in the proximity of locus 44,074,688 (*F*_ST_ = 0.286, Z*F*_ST_ = 3.946, θπ ratio = 2.635) of chromosome 23 and is involved in the hippo signaling pathway, which plays a significant role in controlling follicular growth and ovulation in livestock [56]. *BMP5* belongs to the BMP subfamily and plays a crucial role in ovarian folliculogenesis by enhancing proliferation of granulosa cells, which is associated with the growing female gamete in the mammalian ovary [57], indicating that this gene is more likely to be under selection. *MARF1* and *SF1* polymorphisms are noticeable as they are highly differentiated between the high- and low-reproduction groups under our three approaches used.

Functional enrichment analysis is used to explore a controlled vocabulary to extensively describe the characteristics of genes and gene products. According to our enrichment analysis results, several GO and pathways were directly or indirectly involved with the reproduction of animals, although some of the pathways could not pass the significance threshold (*p* < 0.05). The selected genes were mostly enriched in biological processes, including metabolic processes, regulation of biological processes, developmental processes, and reproductive processes. The most significant GO terms were found binding (GO: 0005488) in molecular functions and intracellular organelles (GO: 0043229) in cellular components. The pathway analysis revealed the involvement of selected genes in reproduction. The mitogen-activated protein kinase (MAPK) pathway has important roles in oocyte maturation and eventually in ovulation by regulating cyclic guanosine monophosphate production and its transportation to the oocyte [58,59]. Other enriched pathways, such as bile secretion (hsa04976), retrograde endocannabinoid signaling (hsa04723), the hippo signaling pathway (hsa04390), the oxytocin signaling pathway (hsa04921), and ovarian steroidogenesis (hsa04913), play significant roles in the processes of oocyte maturation, regulation of follicular growth, and ovulation in livestock [56]. Although some of the candidate genes were enriched in none of the significant pathways, these genes were deemed to be candidate genes for reproduction due to their biological functions and information from published studies.

## 5. Conclusions

In this study, we investigated the patterns of homozygosity and the signature of positive selection in six Chinese goat populations. Demographic decline, the highest level of inbreeding and severe founder effects are the signatures of an increased level of homozygosity in Guangfeng goat while population admixture is associated with the opposite effect in other goat breeds. The existence of two genes in the ROH regions coincided with the regions detected by the selection signal demonstrated that the homozygosity is not solely the result of demography instead of positive selection. Several genes were identified as candidate genes by *F*_ST_, π and XP-EHH algorithm of which *MARF1* and *SF1* genes are highly differentiated between high and low reproduction groups according to the three approaches used. The information about the genetic status and the identified genes under positive selection may be useful for further genetic improvement and setting conservation program for these important goat breeds. Further studies relying on high-density markers are necessary to evaluate the level of genetic autozygosity and signature of selection of the goat breed.

## Figures and Tables

**Table 1:** Single nucleotide polymorphisms (SNPs) filtration result.

Parameters	Goat 50K
Total number of SNPs	51,474
SNP Call frequency (call rate) (<0.95)	1338
Minor allele frequency (<0.05)	2296
Hardy-Weinberg equilibrium (*p* < 1 × 10^−5^)	1814
SNPs removed from sex chromosome	715
Total SNPs removed	6163
Total SNPs remained	45,311

**Table 2:** Descriptive statistics for runs of homozygosity and inbreeding coefficients (F) within each breed. SE, standard error; ***F*_ROH_**, inbreeding coefficient based on runs of homozygosity (ROH); ***F*_HOM_**, Inbreeding coefficient based on the difference between the observed and expected numbers of homozygous genotypes; *R*, correlation; GF, Guangfeng; JG, Jinning Grey; LN, Liaoning; LP, Luoping Yellow; NJ, Nanjiang; and QG, Qinggeli.

Breed	SNP No.	Average Length (Mb)		Average Number		*F*_ROH_			*R* (*F*_ROH_, *F*_HOM_)
		Mean ± SE	Range	Mean ± SE	Range	Mean ± SE	Range	Mean ± SE	
GF	15−1195	3.29 ± 0.001	1.00−64.21	142.29 ± 2.41	53−244	0.19 ± 0.005	0.05−0.48	0.057 ± 0.007	0.97
JG	15−1192	2.5 ± 0.002	1.00−61.64	35.50 ± 0.23	13−76	0.03 ± 0.009	0.006−0.24	−0.002 ± 0.001	0.89
LN	15−1066	2.99 ± 0.000	1.00−55.87	130.26 ± 0.21	91−162	0.162 ± 0.0004	0.105−0.234	−0.034 ± 0.001	0.96
LP	15−1304	1.95 ± 0.001	1.00−67.54	129 ± 0.82	67−154	0.105 ± 0.002	0.03−0.29	0.010 ± 0.003	0.95
NJ	15−580	2.59 ± 0.001	1.16−30.61	69.79 ± 0.63	26−86	0.06 ± 0.0009	0.013−0.117	−0.020 ± 0.001	0.98
QG	15−1158	2.34 ± 0.006	1.00−61.65	26.83 ± 0.45	11−61	0.026 ± 0.001	0.006−0.225	−0.008 ± 0.001	0.99

**Table 3:** Estimated inbreeding levels at each cut-off point of runs of homozygosity.

Breed	*F*_ROH (0–3)_	*F*_ROH (3–5)_	*F*_ROH (5–10)_	*F*_ROH (10–20)_	*F*_ROH (20–30)_	*F*_ROH (>30)_	Mean
GF	0.060167	0.013693	0.015554	0.024719	0.019603	0.026762	0.0267
JG	0.013824	0.002124	0.003958	0.004607	0.003361	0.002482	0.0050
LN	0.053225	0.018406	0.024548	0.02398	0.009217	0.004083	0.0222
LP	0.055294	0.003027	0.004658	0.009596	0.006219	0.007642	0.0144
NJ	0.030963	0.006652	0.006211	0.005015	0.002545	0.000437	0.0086
QG	0.010724	0.001535	0.002563	0.001888	0.002893	0.001974	0.0035

**Table 4:** Reynolds’ genetic distances (above diagonal) and pairwise differences (below diagonal) among four Chinese goat populations.

Breed	GF	JG	LN	LP	NJ	QG
GF		0.06847	0.17687	0.17422	0.11081	0.08963
JG	0.06618		0.11153	0.11506	0.04969	0.02867
LN	0.16211	0.10554		0.20055	0.06391	0.08185
LP	0.15989	0.10869	0.18172		0.13337	0.10824
NJ	0.10489	0.04848	0.06191	0.12486		0.02116
QG	0.08573	0.02826	0.07859	0.10259	0.02094	

**Table 5:** Genomic loci containing the potential candidate genes.

Chromosome	Position	*F*_ST_	Z*F*_ST_	π	XP-EHH	Gene Name
25	13,838,569	0.419	6.129	5.002	3.486	*Meiosis arrest female 1 (MARF1)*
29	43,352,393	0.305	4.262	3.450	2.903	*Steroidogenic factor-1 (SF1)*
24	39,657,370	0.306	4.281	2.409		*Transmembrane Protein 200C (TMEM200C)*
13	55,939,729	0.329	4.647	2.346		*Synaptonemal Complex Protein 2 (SYCP2)*
4	43,874,883	0.301	4.193	2.829		*Adenylate cyclase 1 (ADCY1)*
23	44,074,688	0.286	3.946	2.635		*Bone Morphogenetic Protein 5 (BMP5)*

**Table 6:** List of candidate genes located in genomic regions with a high frequency of ROHs associated with goat reproductive traits.

Breed	CHR	Start (bp)	End (bp)	Gene Symbol	Distance between ROH Region and Gene **	Function
GF	7	63,446,436	66,314,871	GDF9 *	2.57 Mb	Reproduction
GF	11	76,923,989	77,967,512	GDF7 *	0.98 Mb	Reproduction
GF	2	27,707,065	29,287,557	INHA *	0.60 Mb	Reproduction
LP	18	13,400,847	14,834,169	MTHFSD *	0.35 Mb	Reproduction
LP	25	13,070,993	14,141,921	MARF1 *	0.82Mb	Reproduction
GF	13	53,460,986	56,246,114	SYCP2 *	2.48 Mb	Reproduction
JG	24	40,941,530	42,250,672	*TMEM200C*	1.30 Mb	Reproduction
LP	4	41,890,323	42,021,443	*ADCY1*	2.01 Mb	Reproduction

** The distance between genes and ROH regions was calculated by subtracting the starting coordinate of the ROH region from the starting coordinate of the gene; * Candidate genes are located in the ROH region.

## Supplementary Materials

The following supplementary materials are available online at https://www.mdpi.com/2073-4425/10/11/938/s1. Table S1: A short description (Breed name, sample size, fecundity rate, demography and breeding practices, average altitude and agro-ecology) of six goat populations. Table S2: Average sum of ROH content; Table S3: Genomic Regions with a High ROH Frequency; Table S4. Analysis of molecular variance (AMOVA) among six Chinese goat populations; Table S5: Overlapping genes of top 1% *F*_ST_, π ratio and 0.1% XP-EHH; Table S6: GO Term; Table S7: KEGG Pathway.
